# Correlation between geriatric nutritional risk index and intradialytic hypotension in elderly patients undergoing maintenance hemodialysis: a case-control study

**DOI:** 10.1186/s41043-024-00551-1

**Published:** 2024-06-07

**Authors:** Dan Zhang, Zhoushan Fu, Xiaoqin Wan, Xiaojing Wu, Lin Ding

**Affiliations:** Ruijin Hospital, Shanghai Jiaotong University School of Medicine, 888 Shuangding Road, Jiading District, Shanghai, 201801 China

## Abstract

**Background:**

There is a correlation between nutritional status and treatment outcomes and long-term survival in MHD patients but there is limited research on the relationship between GNRI and IDH. This case-control study aimed to investigate the correlation between Geriatric Nutritional Risk Index (GNRI) and intradialytic hypotension (IDH) in elderly patients undergoing maintenance hemodialysis (MHD).

**Methods:**

This study was carried out on 129 cases of MHD patients with IDH and 258 non-IDH-controls in Ruijin Hospital, Shanghai Jiaotong University School of Medicine, China, between June 2020 and May 2022. Professional researchers collected patients’ general information on gender, primary disease, dialysis-related indicators, anthropometric measures, laboratory biochemicals, and GNRI. Logistic regression analysis was used to evaluate the correlation between GNRI and IDH.

**Results:**

A total of 385 elderly MHD patients were included. Compared with GNRI Q4 group, the odds ratios for the risk of IDH in GNRI Q3 group, GNRI Q2 group, and GNRI Q1 group of elderly MHD patients were 1.227, 2.196, and 8.350, respectively, showing a significant downward trend (*P*-trend *<* 0.05). The area under the curve of GNRI for predicting IDH was 0.839 (95% CI: 0.799–0.879). Between different genders, a decrease in GNRI was closely related to an increase in IDH risk (P for trend < 0.05).

**Conclusions:**

This research shows a significant association between GNRI and the incidence of IDH among elderly MHD patients and has an important warning effect. Encouraging the incorporation of GNRI assessment into the clinical assessment protocols of older patients with MHD may help to improve the nutritional status of those suffering from it and reduce the risk of IDH.

## Introduction

End-stage renal disease (ESRD) is a significant public health problem in the whole world, impacting over 1700,000 individuals with an incidence rate of about 0.64% [1]. Maintenance hemodialysis (MHD) is the primary renal replacement therapy modality for ESRD in most countries [2]. However, intradialytic hypotension (IDH) poses a significant and prevalent complication in the context of MHD, with reported incidence rates ranging from 20–30% [3]. In addition, the etiology of IDH is not fully understood, but may involve transient intravascular hypovolemia, impaired compensatory mechanisms, and end-organ hypoperfusion, leading to a higher risk of cardiovascular events and mortality [4, 5]. Notably, elderly MHD patients tend to possess a heightened vulnerability to experiencing IDH, due to advanced age, nutritional status, weight and body mass index related problems and inflammation [6–8]. Given the potential impact of IDH on patient outcomes and treatment outcomes, it is necessary to pay attention to monitoring and addressing IDH during MHD in elderly patients.

GNRI is a valuable nutritional indicator based on serum albumin level and the comparison between actual and ideal body weight, which can effectively evaluate the nutritional status of elderly individuals [9–11]. Research has suggested that there may be a correlation between nutritional risk and the occurrence of IDH in elderly patients undergoing MHD [12]. A lower GNRI score, indicating a higher nutritional risk, may be associated with an increased risk of experiencing IDH during dialysis sessions [13]. This relationship can be attributed to the impact of poor nutritional status on the cardiovascular system and fluid balance, which are key factors in the development of IDH.

Exploring the correlation between GNRI and IDH in elderly MHD patients can provide valuable insights into the role of nutritional status in managing dialysis-related complications and improving outcomes in this vulnerable population. The first aim of the present study is to evaluate the GNRI in elderly patients undergoing MHD. The second aim is to investigate the impact of GNRI on IDH and its predictive value. Specifically, we will evaluate the GNRI in elderly MHD patients and correlate it with subsequent IDH development, aiming to explore the potential role of GNRI as a predictive indicator. The findings of this study will contribute to a better understanding of nutritional assessment and IDH risk evaluation in elderly MHD patients.

## Methods

### Participants

This case-control study was carried out on 129 cases of MHD patients with IDH and 258 non-IDH-controls over 65 years in Ruijin Hospital, Shanghai Jiaotong University School of Medicine, China, between June 2020 and May 2022. The sample size was performed using the PASS Version 11 softwore. Where power = 0.9, α = 0.05, Odds Ratio = 3.4, case-to-control ratio = 1:2, the sample size was initially calculated to be 348. Accommodating 10% non-response rate, a total of 387 patients were recruited for this study consequently.

All MHD patients in this study were dialyzed with deep venous catheters or arteriovenous endovascular fistulas and all were bicarbonate dialysis, and all of them had been receiving MHD treatment for more than 6 months. Experienced nephrologists referred to the expert consensus to diagnose IDH. Specifically, it was accompanied by symptoms of hypotension such as nausea, lethargy, headache, muscle cramps, and chest and back pain, and a drop in systolic blood pressure of ≥ 20 mmHg or a drop in mean arterial pressure of > 10 mmHg on dialysis [14, 15]. The control group was also randomly selected by the same group of nephrologists from our hospital with the same criteria as the case but without IDH and was treated with routine dialysis in the nephrology department. Exclusion criteria were long-term use of hormone or psychiatric drugs (*n* = 1) and bedridden patients (*n* = 1). In the final analyses, a total of 127 cases and 258 controls were included. All research participants understood the purpose and process of the study and provided written consent. This study was approved by Ruijin Hospital, Shanghai Jiao Tong University School of Medicine Ethics Committee approved [Ethics No. (2020) Lin Lun Audit No. (82)].

### Data collection

Professional researchers collected patients’ general information on gender, age, primary disease, comorbidities (hypertension, diabetes mellitus or cardiovascular disease), pre-dialysis antihypertensive medication, dialysis-related indicators, anthropometric measures, laboratory biochemicals, and GNRI.

### Anthropometric indicators

Participants’ weight and height were measured by standardized methods using an electronic digital scale (accuracy 100 g) and a telemeter (accuracy 0.5 cm), respectively. Body mass index (BMI) was reported using the following formula: BMI = weight/height^2^ (kg/m^2^).

### Dialysis-related indicators

Food intake during MHD, age on dialysis, pre-dialysis systolic blood pressure, weight gain between MHD, ultrafiltration rate, ultrafiltration volume, and urea clearance (URR) were captured through the electronic medical record system.

### Laboratory biochemical indicators

The results of the most recent biochemical indices at the time of IDH were captured through the electronic medical record system, including erythrocyte distribution width, albumin, mean erythrocyte volume, hemoglobin, parathyroid hormone (iPTH), blood sodium, blood calcium, blood creatinine, triglycerides, urea nitrogen, cholesterol, and low-density lipoprotein (LDL-C).

### Calculation of GNRI

To calculate the GNRI, we used the formula as follows [1.489 × albumin (g/L)] + [41.7 × (actual weight/ideal weight)] [9]. The actual weight was the weight (kg) measured by an electronic digital scale, and the ideal weight determined using the following formula: the ideal weight (kg) = 22 × actual height^2^ [9].

### Statistical analysis

Statistical analysis was performed using SPSS 22.0 software. Continuous variables with non-normal distribution were represented as median (P25, P75) and analyzed using non-parametric tests. Continuous variables with a normal distribution were presented as mean ± standard deviation $(\overline x \pm s)$ and analyzed using independent sample t-test. Categorical variables were presented as n (%) and analyzed using appropriate tests. Logistic regression analysis was performed to assess the correlation between GNRI and IDH in elderly MHD patients. Logistic analysis was performed in our study using three models. No confounders were adjusted in Model (1) Age and dialysis vintage were adjusted in Model (2) Age, dialysis vintage, BMI, oral intake, mean corpuscular volume, albumin, blood creatinine, iPTH, systolic blood pressure pre-dialysis, interdialytic weight gain, ultrafiltration rate, and pre-dialysis antihypertensive drugs use were adjusted in Model (3) A *P*-value < 0.05 was considered statistically significant for differences.

## Results

A total of 385 elderly MHD patients were included in the study. The general characteristics of participants between groups are summarized in Table 1. Compared to the controls, the cases had statistically significant differences in age, BMI, dialysis vintage, mean corpuscular volume, albumin, GNRI, blood creatinine, iPTH, systolic blood pressure pre-dialysis, interdialytic weight gain, ultrafiltration rate, proportion of oral intake, and proportion of taking antihypertensive drugs before dialysis (*P <* 0.05).

**Table 1:** General characteristics of participants between groups

	N (%), Mean ± SD or Median (P25, P75) Cases (*n* = 127)	N (%), Mean ± SD or Median (P25, P75) Controls (*n* = 258)	*p*-value
Gender			
Male	67 (52.76)	158 (61.24)	0.112
Female	66 (47.24)	100 (38.76)	
Age (years)	70.87 ± 8.93	67.83 ± 8.33	0.001
BMI (kg/m^2^)	19.87 ± 4.20	21.09 ± 3.73	0.004
Primary diseases			0.145
Hypertensive nephropathy	40 (31.50)	94 (36.43)	
Diabetic nephropathy	38 (29.92)	50 (19.38)	
Chronic glomerulonephritis	29 (22.83)	69 (26.74)	
Other etiologies	20 (15.75)	45 (17.44)	
Hypertension			0.241
No	91 (71.65)	199 (77.13)	
Yes	36 (28.35)	59 (22.87)	
Diabetes			0.127
No	72 (56.69)	167 (64.73)	
Yes	55 (43.31)	91 (35.27)	
Cardiovascular disease			0.245
No	56 (44.09)	130 (50.39)	
Yes	71 (55.91)	128 (49.61)	
Dietary intake during MHD			< 0.001
No	74 (58.27)	198 (76.74)	
Yes	53 (41.73)	60 (23.26)	
Duration of dialysis (years)	6.10 (4.60, 7.80)	5.00 (3.50, 6.40)	< 0.001
Red blood cell distribution width (%)	14.50 ± 2.74	14.06 ± 2.66	0.136
Mean corpuscular volume (fl)	79.52 ± 11.40	83.17 ± 13.12	0.008
Albumin (g/L)	36.57 (32.90, 40.56)	40.72 (35.83, 45.92)	< 0.001
Hemoglobin (g/L)	89.46 ± 17.53	92.76 ± 19.27	0.105
Sodium (mmol/L)	138.94 ± 16.51	141.76 ± 18.92	0.153
Calcium (mmol/L)	2.21 ± 0.57	2.29 ± 0.62	0.223
GNRI	89.35 (85.01, 94.74)	99.54 (94.30, 104.71)	< 0.001
Creatinine (µmol/L)	827.00 (710.00, 981.00)	924.00 (775.25, 1056.25)	< 0.001
Blood urea nitrogen (mmol/L)	27.94 ± 6.51	29.02 ± 7.38	0.162
Triglycerides (mmol/L)	1.63 ± 0.45	1.72 ± 0.53	0.101
Cholesterol (mmol/L)	4.01 ± 0.94	4.18 ± 1.09	0.134
LDL-C (mmol/L)	2.50 ± 0.57	2.58 ± 0.39	0.107
iPTH (pg/mL)	439.44 (426.84, 454.77)	416.66 (405.55, 436.54)	< 0.001
Systolic blood pressure pre-dialysis (mmHg)			0.014
<110	13 (10.24)	8 (3.10)	
110–139	38 (29.92)	79 (30.62)	
≥ 140 (247)	76 (59.84)	171 (66.28)	
Weight gain during MHD (%)	3.28 (2.8, 4.12)	3.07 (2.23, 3.55)	< 0.001
Ultrafiltration rate (mL/kg/h)	14.02 ± 3.84	12.61 ± 3.93	0.001
Ultrafiltration volume (L)	3.30 ± 0.93	3.18 ± 0.84	0.184
URR (%)	63.53 ± 13.34	65.11 ± 11.92	0.235
Taking antihypertensive drugs before dialysis			0.015
No	86 (67.72)	204 (79.07)	0.015
Yes	41 (32.28)	54 (20.93)	

BMI = body mass index; GNRI = geriatric nutritional risk index; LDL-C = low-density lipoprotein; iPTH = intact parathyroid hormone; MHD = maintenance hemodialysis; URR = urea clearance rate

Based on the quartiles of GNRI in elderly MHD patients, the patients were divided into four groups: Q1 group (≤ 90.28), Q2 group (90.29–96.14), Q3 group (96.15-102.76), and Q4 group (≥ 102.77). There were statistically significant differences (*P<*0.05) among different GNRI groups in terms of age, BMI, dietary intake during MHD, duration of dialysis, systolic blood pressure pre-dialysis, weight gain between MHD sessions, ultrafiltration rate, albumin, iPTH, and serum creatinine. General characteristics of participants across quartiles of GNRI are presented in Table 2.

**Table 2:** General characteristics of participants across quartiles of geriatric nutritional risk index (GNRI)

	N (%), Mean ± SD or Median (P25, P75) Q1 group (*n* = 96)	N (%), Mean ± SD or Median (P25, P75) Q2 group (*n* = 97)	N (%), Mean ± SD or Median (P25, P75) Q3 group (*n* = 96)	N (%), Mean ± SD or Median (P25, P75) Q4 group (*n* = 96)	*p*-value
Gender					0.228
Male	48 (50.00)	52 (53.61)	60 (62.50)	59 (61.46)	
Female	48 (50.00)	45 (46.39)	36 (37.50)	37 (38.54)	
Age (years)	72.75 ± 8.31	69.44 ± 9.52	68.25 ± 7.94	66.89 ± 6.78	< 0.001
BMI (kg/m^2^)	18.91 ± 4.06	19.82 ± 3.75	21.67 ± 3.69	22.36 ± 3.22	< 0.001
Primary diseases					0.991
Hypertensive nephropathy	30 (31.25)	34 (35.05)	36 (37.50)	34 (35.42)	
Diabetic nephropathy	26 (37.08)	23 (23.71)	19 (19.79)	20 (20.62)	
Chronic glomerulonephritis	24 (25.00)	24 (24.74)	25 (26.04)	25 (25.77)	
Other etiologies	16 (16.67)	16 (16.49)	16 (16.67)	17 (17.53)	
Hypertension					0.947
No	74 (77.08)	72 (74.23)	72 (75.00)	72 (75.00)	
Yes	22 (22.92)	25 (25.77)	24 (25.00)	24 (25.00)	
Diabetes					0.859
No	59 (61.46)	58 (59.79)	59 (61.46)	63 (65.63)	
Yes	37 (38.54)	39 (40.21)	37 (38.54)	33 (34.38)	
Cardiovascular disease					0.797
No	49 (51.04)	43 (44.33)	48 (50.00)	46 (47.92)	
Yes	47 (48.96)	54 (55.67)	48 (50.00)	50 (52.08)	
Dietary intake during MHD					0.024
No	56 (58.33)	73 (75.26)	71 (73.96)	72 (75.00)	
Yes	40 (41.67)	24 (24.74)	25 (26.04)	24 (25.00)	
Duration of dialysis (years)	5.95 (4.30, 7.30)	5.80 (4.05, 6.80)	4.65 (3.50, 6.30)	5.05 (3.70, 6.50)	0.003
Red blood cell distribution width (%)	14.27 ± 2.73	14.12 ± 2.68	14.16 ± 2.75	14.27 ± 2.64	0.973
Mean corpuscular volume (fl.)	81.46 ± 12.53	80.95 ± 11.26	81.27 ± 13.26	84.19 ± 13.49	0.261
Albumin (g/L)	36.75 (31.48, 40.01)	38.88 (33.47, 41.42)	40.72 (35.22, 44.20)	46.04 (39.04, 48.23)	< 0.001
Hemoglobin (g/L)	89.51 ± 17.20	92.53 ± 15.48	88.75 ± 19.64	93.36 ± 16.26	0.179
Sodium (mmol/L)	138.15 ± 17.02	141.03 ± 19.52	137.34 ± 14.61	142.18 ± 18.25	0.173
Calcium (mmol/L)	2.19 ± 0.47	2.24 ± 0.63	2.28 ± 0.51	2.31 ± 0.56	0.457
Creatinine (µmol/L)	799.00 (688.75, 911.25)	902.00 (748.50, 1009.00)	950.50 (773.75, 1045.50)	1019.50 (823.50, 1176.00)	< 0.001
Blood urea nitrogen (mmol/L)	27.53 ± 6.24	29.06 ± 7.11	29.43 ± 7.58	28.51 ± 6.47	0.245
Triglycerides (mmol/L)	1.59 ± 0.42	1.62 ± 0.56	1.71 ± 0.45	1.73 ± 0.52	0.138
Cholesterol (mmol/L)	4.03 ± 1.06	3.92 ± 0.86	4.15 ± 1.12	4.23 ± 0.92	0.146
LDL-C (mmol/L)	2.46 ± 0.57	2.51 ± 0.58	2.61 ± 0.35	2.53 ± 0.41	0.197
iPTH (pg/mL)	434.84 (418.07, 450.21)	429.36 (411.47, 446.55)	423.68 (408.49, 439.34)	427.08 (407.96, 438.06)	0.001
Systolic blood pressure pre-dialysis (mmHg)					0.042
<110	7 (7.29)	10 (10.31)	1 (1.04)	3 (3.13)	
110–139	31 (32.29)	21 (21.66)	34 (35.42)	31 (32.29)	
≥ 140	58 (60.42)	66 (68.04)	61 (63.54)	62 (64.58)	
Weight gain during MHD (%)	3.40 ± 0.92	3.06 ± 0.89	2.99 ± 0.82	2.96 ± 0.83	0.001
Ultrafiltration rate (mL/kg/h)	13.72 ± 3.66	13.37 ± 3.82	12.12 ± 3.96	13.09 ± 4.23	0.032
Ultrafiltration volume (L)	3.27 ± 0.93	3.09 ± 0.78	3.24 ± 0.85	3.26 ± 0.92	0.418
URR (%)	62.73 ± 14.61	66.42 ± 11.52	64.28 ± 10.94	64.86 ± 12.78	0.229
Taking antihypertensive drugs before dialysis					0.584
No	71 (73.96)	73 (75.26)	77 (80.21)	69 (71.88)	
Yes	25 (26.04)	24 (24.74)	19 (19.79)	27 (28.13)	

BMI = body mass index; GNRI = Geriatric Nutritional Risk Index; iPTH = intact parathyroid hormone; LDL-C = low-density lipoprotein; MHD = maintenance hemodialysis; URR = urea clearance rate

The correlation between IDH and GNRI is shown in Table 3. The decrease in GNRI was correlated with an increased risk of IDH. After adjusting for age, dialysis vintage, BMI, oral intake, mean corpuscular volume, albumin, blood creatinine, iPTH, systolic blood pressure pre-dialysis, interdialytic weight gain, ultrafiltration rate, and pre-dialysis antihypertensive drugs use, the results revealed that compared to the GNRI Q4 group, the GNRI Q3 group, GNRI Q2 group, and GNRI Q1 group of elderly MHD patients had odds ratios (OR) for the risk of IDH with values of 1.227, 2.196, and 8.350, respectively. These increasing trends were statistically significant (*P <* 0.05). Notably, the Q1 group (OR = 8.350, 95%CI: 3.045–22.901) exhibited a significantly higher risk of IDH compared to the GNRI Q4 group (*P <* 0.05). The results of the multivariable logistic regression analysis are presented in Table 3.

**Table 3:** Logistic regression of the correlation between intradialytic hypotension (IDH) and geriatric nutritional risk index (GNRI)

	OR (95%CI)		
	Mode 1	Mode 2	Mode 3
GNRI			
Q4	Ref	Ref	Ref
Q3	0.720 (0.353–1.467)	0.831 (0.396–1.744)	1.227 (0.445–3.382)
Q2	2.223 (1.199–4.122)	2.109 (1.097–4.058)	2.196 (0.860–5.605)
Q1	6.825 (3.622–12.861)	6.270 (3.134–12.545)	8.350 (3.045–22.901)
*P*-tend	< 0.001	< 0.001	< 0.001

Model 1: Crude. Model 2: adjusted for age and dialysis vintage. Model 3: adjusted for age, dialysis vintage, BMI, oral intake, mean corpuscular volume, albumin, blood creatinine, iPTH, systolic blood pressure pre-dialysis, interdialytic weight gain, ultrafiltration rate, and pre-dialysis antihypertensive drugs

## Discussion

In this case-control study, low nutritional status measured using the GNRI was positively associated with the odds of IDH. These associations remained significant after adjusting for several confounding variables, including age, age on dialysis, BMI, food intake, mean red blood cell volume, albumin, blood creatinine, iPTH, pre-dialysis systolic blood pressure, weight gain between MDHs, ultrafiltration rate, and pre-dialysis antihypertensive medication use. This study is the first to date to investigate the association between GNRI and the odds of IDH in an elderly MHD population.

In individuals undergoing MHD, key nutritional parameters such as muscle mass, body weight, and serum albumin level invariably demonstrate notable declines. These declines are magnified with the extension of dialysis treatment duration. These reductions may be attributed to a constellation of factors: gastrointestinal symptoms, derangements in protein-energy metabolism, infectious processes, and detrimental impacts engendered by uremic toxins, cumulatively precipitating a downturn in nutritional intake [12, 16]. Although there has been some correlation between nutritional status and treatment outcome and long-term survival in patients with MHD in previous studies [17], no study has evaluated the relationship between GNRI and the risk of IDH. In the present study, the risk of IDH trended upward with decreasing GNRI, where participants in the lower quartile of GNRI were more than 7 times more likely to develop IDH compared with participants in the highest quartile of GNRI. GNRI may influence patient survival outcomes through IDH, further enriching the use of GNRIs in elderly patients with MHD. Elderly patients with MHD are more prone to IDH due to poor nutritional status, low physiological reserve, and reduced effective blood volume, which significantly impacts the dialysis process and increases the difficulty of nursing and treatment [18, 19]. Therefore, healthcare professionals should pay more attention to monitoring the nutritional status of elderly MHD patients.

GNRI is a widely used evaluation tool in clinical practice, and its value in nutritional assessment has been confirmed in numerous studies [16, 20]. In this study, 385 elderly MHD patients were included, with an average GNRI of 96.26 ± 7.91 (78.92-112.56), slightly higher than that reported by Nouri et al. [20]. GNRI is calculated based on factors such as serum albumin and body weight. The analysis results of this study revealed significant differences in age, dialysis duration, BMI, serum albumin, blood creatinine, iPTH, systolic blood pressure pre-dialysis, interdialytic weight gain, and ultrafiltration rate among different GNRI groups in elderly MHD patients. These differences may be attributed to the influence of nutritional status on hormone metabolism, the dialysis process, and blood pressure changes [21]. What’s more, gender stratification analysis showed that a decrease in GNRI and an increase in IDH risk were closely related between different genders. GNRI is calculated based on weight and serum albumin level, both of which reflect reduced protein reserves and decreased physical function, making patients more susceptible to abnormal blood pressure fluctuations [22, 23]. Our findings suggest that GNRI is closely related to the occurrence of IDH in elderly MHD patients and has an important warning effect. The following preventive interventions can be implemented [24, 25]: (1) reduce or avoid the use of antihypertensive drugs on the day of dialysis; (2) control dietary intake during dialysis to limit dry weight gain and regularly monitor dry weight; (3) continuously measure patients’ blood pressure during dialysis to promptly detect or predict the occurrence of IDH; (4) calculate the patient’s GNRI before dialysis; (5) consider extending the duration of dialysis, and ultimately reduce the risk of IDH.

In addition, independent risk factors for IDH include dietary intake, duration of dialysis, iPTH levels, interdialytic weight gain, administration of antihypertensive drugs before dialysis, mean corpuscular volume, albumin level, and systolic blood pressure pre-dialysis. These findings are consistent with previous studies [3, 17]. However, the International Society for Renal Nutrition and Metabolism has reached a consensus stating that the provision of nutrition during the process of hemodialysis can enhance the individual’s nutritional status, and ultimately contribute to improved survival rates [26]. Therefore, further clinical validation and consideration of other clinical factors are necessary for the impact of dietary intake on IDH.

This study is retrospective in nature, and although multiple confounding factors have been considered, there are still some missing data, including prealbumin and protein intake, which were not included in the analysis and may affect the predictive accuracy of GNRI for IDH. Furthermore, this study is a single-center study with a limited sample size. Therefore, further large-scale prospective studies are needed to validate the conclusions.

## Conclusions

Our research shows a significant association between GNRI and the incidence of IDH among elderly MHD patients and has an important warning effect. Its straightforward calculation methodology and ready accessibility render GNRI a practical tool for healthcare practitioners engaged in the risk assessment of elderly MHD patients, both preemptively and during hemodialysis sessions. This risk stratification capability enables timely implementation of targeted interventions, thereby mitigating the prevalence of IDH episodes. Despite certain limitations inherent in our study, the observed correlation between GNRI and IDH underscores the importance of integrating GNRI assessment into the clinical evaluation protocol for elderly MHD patients, thus facilitating informed decision-making and personalized management strategies.

## Data Availability

The data used for this project are available upon reasonable request to the corresponding author.
